# Embolism does not spread outwards from simulated herbivory damage sites in leaves

**DOI:** 10.1093/aobpla/plag033

**Published:** 2026-08-24

**Authors:** Lisa Sarah García, Ilaíne Silveira Matos, Todd Dawson, Benjamin Wong Blonder

**Affiliations:** Department of Environmental Science, Policy, and Management, University of California at Berkeley, 130 Mulford Hall #3114, Berkeley, CA 94720, United States; Department of Biology, MSC03-2020, 1 University of New Mexico, Albuquerque, NM 87131, United States; Graduate Degree Program in Ecology, Colorado State University, 200 W. Lake St., Fort Collins, CO 80523, United States; Department of Environmental Science, Policy, and Management, University of California at Berkeley, 130 Mulford Hall #3114, Berkeley, CA 94720, United States; School of Biological Sciences, Adelaide University, Adelaide, SA 5005, Australia; Department of Environmental Science, Policy, and Management, University of California at Berkeley, 130 Mulford Hall #3114, Berkeley, CA 94720, United States; Department of Integrative Biology, University of California at Berkeley, Valley Life Sciences Building #3140, Berkeley, CA 94720, United States; Department of Environmental Science, Policy, and Management, University of California at Berkeley, 130 Mulford Hall #3114, Berkeley, CA 94720, United States

**Keywords:** herbivory, hydraulic vulnerability, leaf venation, embolism

## Abstract

Embolism is known to occur in leaf venation networks as the leaf dries out. This process is associated with reduced leaf hydraulic conductance that can impact transpiration and photosynthesis. We hypothesize that herbivory or other leaf mechanical damage could introduce additional air into the leaf, leading to further venation-mediated embolism spread during drought. Using the optical vulnerability method, we compared embolism spatial spread rates after either midrib or lamina damage in two species of contrasting venation architecture (pinnate vs. palmate). We then tested the hypothesis that embolism would spread faster near damage locations. We found that there was no consistent effect of damage but rather substantial spatial heterogeneity in how damage influences leaf hydraulic conductance. Herbivory damage and hydraulic damage may have independent effects in the studied species.

## Teaser

Embolism is known to appear in leaf venation networks during desiccation. This process can reduce leaf hydraulic conductance that can impact transpiration and photosynthesis. Our study investigated the response of two different venation architectures (pinnate and palmate) to simulated herbivory to the midrib and leaf lamina. We tested if embolism would spread faster from the damage sites than undamaged sites. Our study showed no consistent effect of damage, but substantial species and spatial heterogeneity in how damage influences leaf hydraulic conductance. These findings expand knowledge on potential relationships between herbivory resistance and drought tolerance.

## Introduction

Understanding how damage to venation networks affects leaf capacity to transport water (and therefore transpiration and photosynthesis) is key for modelling plant performance in different environments. It is well known that drought damage promotes embolism, i.e. the formation and propagation of air bubbles inside the leaf veins, which initiate in the larger veins (e.g. midrib) and progressively spread to the smaller conduits as the leaf dries out (Scoffoni *et al.* 2017b). However, local spreading of embolism may occur not only in response to drought but also when herbivores damage leaves (Guan *et al.* 2021). If not sealed immediately (e.g. via tyloses), the veins damaged by herbivores could act as entry points for air, thus potentially introducing embolism into the venation system in locations that might not ordinarily embolize first (e.g. smaller veins; Guan *et al.* 2021). Given that most leaves, particularly in tropical environments, are exposed to some degree of herbivory, studying drought vulnerability only in undamaged leaves is a critical limitation in the current literature (Adams *et al.* 2009, Turcotte *et al.* 2014).

**Figure 1:**
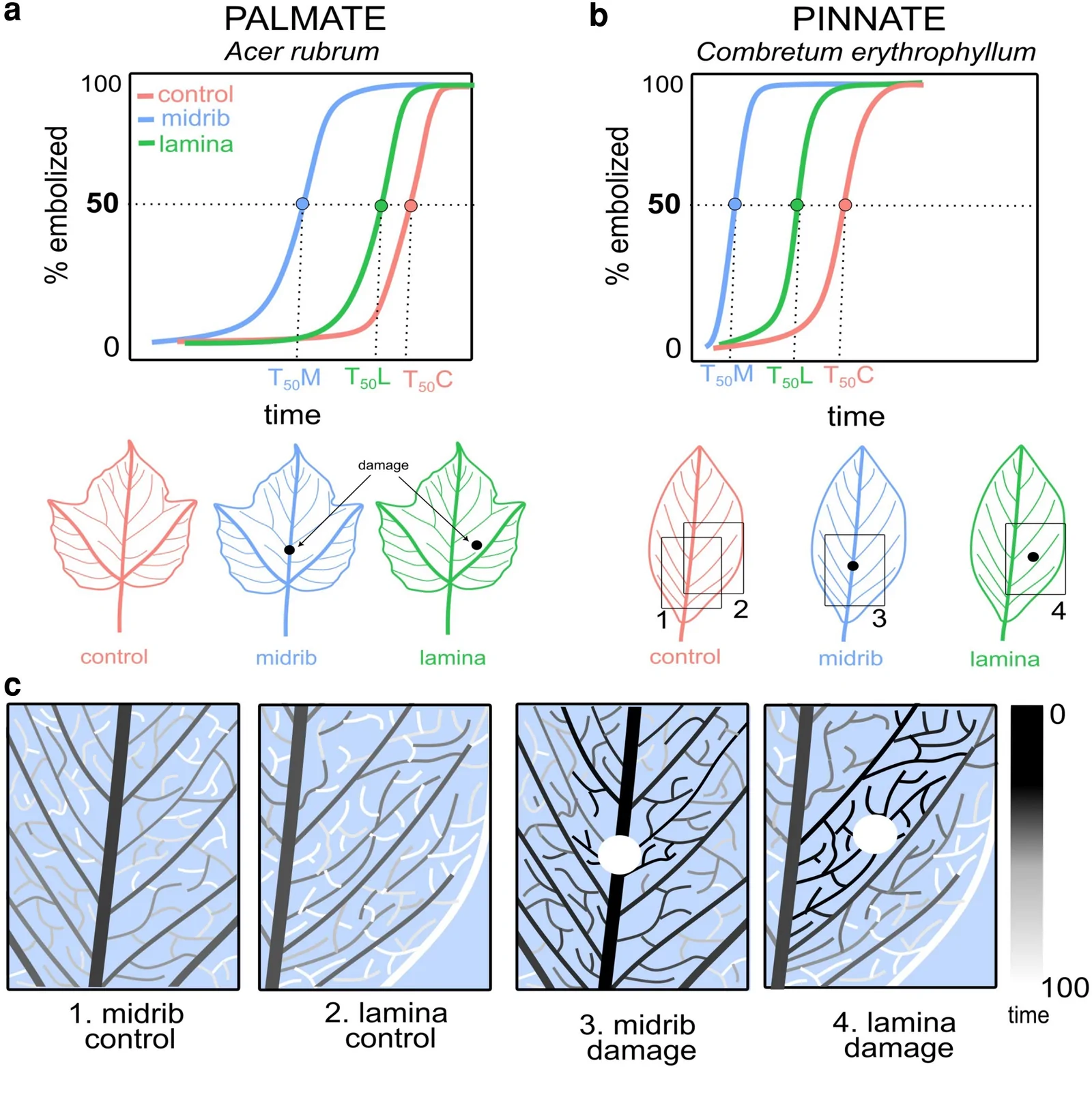
Illustration of the hypothesis tested in this study. (a and b) Show the shape of vulnerability curves (% embolized pixels vs. time) expected for each treatment (control—no damage, midrib damage, lamina damage) and species (*A. rubrum*: palmate leaves and *C. erythrophyllum:* pinnate leaves). We expect the pinnate leaf to show higher vulnerability than the palmate one and midrib and lamina to exhibit higher embolism rates (lower T50) than the control treatment. (c) Shows the expected spatial pattern of embolism spread for equivalent-sized areas in each treatment for the pinnate species. We expect a higher number of embolism to initiate in the proximity of the damaged areas, particularly around the midrib damage.

Presently, it is unclear how drought and herbivory damage interact to dictate the spatio-temporal patterns of embolism propagation and the whole leaf hydraulic vulnerability. The most widely used approach to describe leaf vulnerability to embolism is to construct vulnerability curves (Fig. 1) and extract the P50 [xylem water potential inducing 50% loss of hydraulic conductance (Brodribb *et al.* 2016b)] and/or T50 parameters [time required to embolize 50% of xylem conduits (Brodribb *et al.* 2016a)]. Species with more negative P50 and/or larger T50 are considered less vulnerable to embolism under drought. An important question is whether damage to the venation network, e.g. by herbivores or xylem-feeding insects, can introduce extra embolism via the air-cut conduits (Guan *et al.* 2021, Avila *et al.* 2022), thus altering these vulnerability parameters. Artificial introduction of air bubbles when stem xylem tissue is cut open is a well-known methodological issue in plant hydraulic studies (i.e. excision artefacts [Sperry 2013, Wheeler *et al.* 2013]). But it remains elusive whether cutting sections of the venation network under atmospheric pressure in dehydrating leaves can have a similar effect, leading to reduced embolism resistance and faster embolism propagation (i.e. smaller T50). While Scoffoni and Sack (2015) reported that the excision artefact does not impact leaf xylem hydraulic conductance, Guan *et al.* (2021) clearly showed the formation of extra embolism around open-cut minor veins. As far as we know, no previous study has investigated the impact of major vein damage on embolism propagation under drought.

The magnitude of drought-herbivory combined impact on embolism spreading could be mediated by both the type of herbivory damage and the type of venation network. Features of the venation network architecture could either facilitate or hamper the propagation of embolism after herbivory-induced damage. For example, previous studies have shown that vascular redundancy in the network, particularly in the larger veins, can confer higher tolerance to vein breakage (Sack *et al.* 2008). This explains why damages to the midrib tend to have larger hydraulic impacts in pinnate (single large vein) than in palmately veined leaves (multiple large veins), as in the latter venation type the water can continue to flow around the damaged areas via the redundant larger veins (Sack *et al.* 2008). Contrastingly, vein redundancy can potentially increase damage impact by promoting a faster spread of embolism further away from the damaged areas (Duarte *et al.* 2023). Therefore, the net effect of venation redundancy on the leaf response to herbivory damage is still unclear (Matos *et al.* 2024). Differences in the spatial patterns of leaf consumption by distinct groups of herbivores may also impact the leaf responses to drought-herbivory damage (Malishev and Sanson 2015). While many insect herbivores avoid consumption of the tough leaf midrib tissues, others exhibit plant defence sabotage behaviours—such as girdling, furrowing, and canal cutting—and preferentially sever the midrib and other large-sized veins (Portillo-Estrada and Niinemets 2018). Given that larger veins are crucial for long-distance water flow across the leaf and are more prone to air-seeding (Scoffoni *et al.* 2017b), attacks to the midrib in drought-stressed leaves could lead to disproportionately larger hydraulic vulnerability compared to damage to leaf lamina (i.e. smaller veins). But we lack experimental evidence to support this hypothesis.

In this study, we used the optical vulnerability method (Brodribb *et al.* 2016a, 2016b) to investigate the interactive effects of drought and herbivory damage on the formation and propagation of leaf embolism in two tree species with contrasting venation architectures (Fig. 1)—the palmately veined *Acer rubrum* L. (Fig. 1a) and the pinnately veined *Combretum erythrophyllum* (Burch.) Sond. (Fig. 1b). We were interested in investigating how the spatio-temporal patterns of embolism propagation may differ depending on the type of primary venation architecture (pinnate vs. palmate) and the type of herbivory damage (midrib vs. lamina). Specifically, we investigated the following questions and hypotheses:

- (Q1) Is the timing of spatial embolism spread influenced by the leaf primary vein architecture (pinnate vs. palmate)? (H1) Embolism will spread faster (lower T50) in the pinnate network, because it has fewer larger veins to resist the drought plus herbivory damage.
- (Q2) Does embolism spread faster when the herbivory damage affects a midrib (larger veins) versus the leaf lamina (smaller veins)? (H2) Damage to midrib will result in faster embolism spreading (lower T50) because air-seeding events can propagate further distances through the lower-resistant (i.e. larger) conduits within the midrib, while damage to the lamina will have localized spreading due to the short dimensions of minor veins.
- (Q3) For equivalent-sized areas, is the rate of embolized pixels versus time affected by the proximity to the herbivory-damaged areas? (H3) Embolism will spread faster (lower T50) near herbivory-damaged sites because there will be more veins directly exposed to air to initiate embolism.

## Materials and methods

### Study species

The two studied species with contrasting venation patterns—*C. erythrophyllum* (Combretaceae) and *A. rubrum* (Sapindaceae) were collected at the University of California at Berkeley Botanical Gardens (37° 52′ 11.99″ N −122° 13′ 48″ W) and central campus (37° 52′ 11.99″ N −122° 15′ 0″ W), respectively. These species were selected because they have quite distinct venation types—pinnate versus palmate—and relatively thin and large leaves, which made them particularly suitable for the determination of embolism propagation via the optical method. Nine branches, each ∼1 m long, similar in basal diameter (1–1.5 cm), and a similar number of leaves (in the same species’ replicates), were used for each species. Standardization of branch sizes reduces the possibility that variation in branch capacitance or rate of water loss would be different within species. However, *C. erythrophyllum* branches had more leaves per branch than *A. rubrum* (Supplementary Fig. S7). Branches were collected in July and August 2021, around 10 a.m. and immediately recut under water, then rehydrated for 24 hours in the dark prior to the experiments. Samples were collected from a single individual per species, as our study focused on the interspecific, rather than intraspecific differences, in the spatio-temporal patterns of embolism propagation.

### Drought and simulated herbivory experiments

For each branch, a mature, damage-free, and sun-exposed leaf at the top of the branch was selected and subjected to one of the three treatments (control, lamina, or midrib). In the midrib treatment, a 1.5-mm diameter hole was punched (with a hand punch tool with a 5.1- cm reach) at one-third of the distance from the leaf base (Fig. 1). In the lamina treatment, the hole was punched at the same distance from the leaf base and at 1 cm from the midrib, always on the right side of the leaf. All other mature leaves on the sample branch were also punched (either on the lamina or on the midrib, depending on the treatment type), to elicit any physiological responses to herbivory (e.g. releasing of volatile signals [Portillo-Estrada and Niinemets 2018]). In the control treatment, no leaves were damaged.

The selected leaves (including control leaves) were carefully placed between two glass slides and taped onto a scanner (Epson Perfection V850 Pro) for determination of embolism formation and spread using the optical method (Brodribb *et al.* 2016b). To prevent the branches from being accidentally moved during the scanning process, we hung and firmly taped all branches in an upside-down position above the scanners. Three leaves per species per treatment were scanned, totalling 18 whole-leaf scanned sequences. Leaves were scanned every 15 minutes at 4800 dpi until leaves were dry and crispy. Leaf water potentials were measured using a pressure chamber (PMS, Inc., Model 1000) every 10 minutes when the experiment first started. After 6 hours, we reduced measurement intervals to every 30 minutes until the end of the experiment. Unfortunately, most water potential data did not capture values in the range of −4 to −6 MPa. In this range, the instrument's grommet began to leak or tear. We did not have the time and resources to repeat all the experiments; our data were therefore not suitable for construction of vulnerability curves, so P50 (the water potential at which 50% conductivity is lost) was not investigated in this study.

### Mapping embolism propagation

All scanned images were processed using the software ImageJ (ImageJ 1.54f), following the protocol described in github.com/OpenSourceOV/image-processing-instructions, with some modifications, as described below. This allowed us to identify single embolism events based on differences in pixel brightness, as the light from the scanner shines differently through xylem vessels that are filled with water versus air (Brodribb *et al.* 2016a). Images were rescaled to 50% of original size to address computer memory limitations. All embolism events were identified manually based on visual differences from the previous and following image, to ensure events were not noise. Some minor image registration issues occurred due to continued shrinkage of the leaf during dehydration (e.g. Supplementary Fig. S1, lamina treatment Replicate #3) but were assumed to not impact the analyses.

To construct the vulnerability curves as percentage of embolized pixels versus time, we first used ImageJ to delineate a region-of-interest image for each leaf sample, providing a mask of locations that contained leaf lamina and that were not obscured by tape or other imaging artefacts. The stack of embolism events was then masked to the region-of-interest. Next, we used the LeafVeinCNN programme (version 2.14.00 [Xu *et al.* 2021]) to segment vein versus non-vein pixels in the first scanned image for each leaf. Parameters varied for each sample (‘Tile’ of 10–150, ‘Patch’ from 150–200, thresholds via Otsu algorithm, see Xu *et al.* 2021 for parameter definitions) and were selected by hand to yield the most accurate output. This image was then also masked to the region of interest. A Euclidean distance transformation was applied to identify vein radii for each vein segmented.

For whole-leaf analysis, we then calculated the number of embolized pixels in each image frame. The cumulative sum of this time series was then inferred and divided by the total sum of embolized pixels. The T50 parameter (time to reach 50% of embolized pixels) was extracted from each of the vulnerability curves of cumulative embolized pixels versus time. For each leaf, we also calculated the total fraction of leaf area embolized, by dividing the total sum of embolized pixels by the total number of pixels in the region-of-interest. To visualize the spatial dynamics of embolization, colour-coded maps showing the spatio-temporal sequence of embolism spreading were generated for each sample, following github.com/OpenSourceOV/image-processing-instructions.

**Figure 2:**
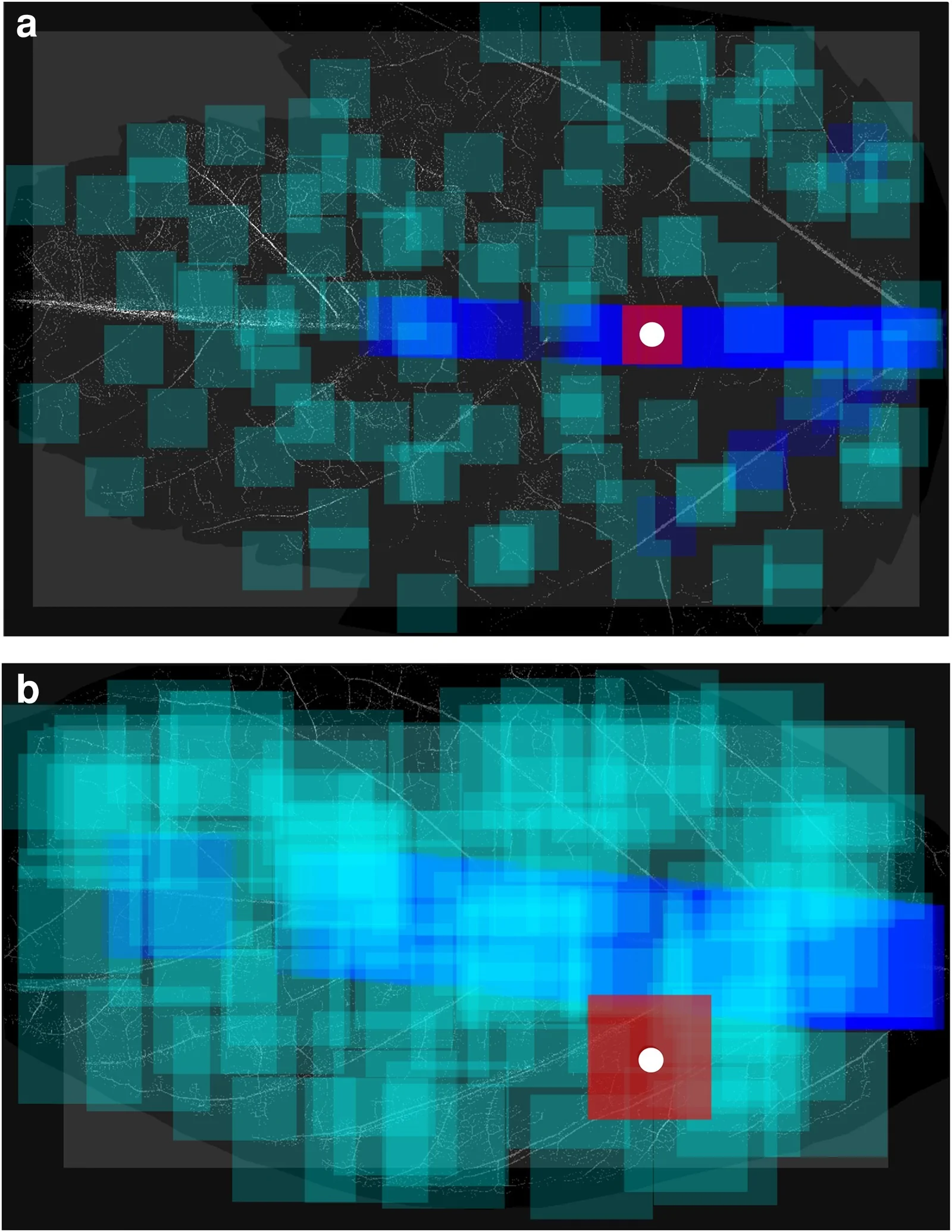
Example of the spatial sampling approach to investigate embolism spreading around and far away from damaged areas. Square sampling regions are shown for the focal damage location (red; note the circular punch-out of area inside the square), random locations within the non-damaged midrib (blue, ‘random_big’; same terminology in Fig. 4), and random locations within the non-damaged lamina (cyan, ‘random_small’). Note the Euclidean distance transformation approach yielded one false-positive midrib inference in the image top-right, which was deleted from the analysis. (a) Example for *A. rubrum* (palmate) leaf in the midrib treatment (second leaf replicate), showing 200 pixel sized squares. Embolism is shown in grey (cropped to region of interest). (b) Example for *C. erythrophyllum* (pinnate) leaf in the lamina treatment (second leaf replicate), showing 400 pixel sized squares.

To investigate potential differences in the spatio-temporal dynamics of embolization between areas located nearby versus far away from the damaged points, we first randomly selected 200 square regions, of which 100 were located within the leaf lamina areas with minor veins (‘random_small’, i.e. with veins <4 pixels in radii) and 100 were located within the leaf midrib (‘random_big’, i.e. with veins >12 pixels in radii) (see Fig. 2). To test if the size of the focal area would influence the analysis, we tested square regions with either 200 or 400 pixel side length. The centre coordinates were selected to be within the region of interest, sufficiently far away from the image boundary, and within vein pixels. We also selected a focal square region of the same size corresponding to the location of the lamina or midrib damage (no focal region was selected in control leaves).

In each square region, we constructed a time series of the cumulative sum of the number of embolized pixels (allowing for the possibility that the same pixel might embolize more than once during the experiment). These values were normalized in two different ways: (i) by the total number of pixels in each square, after discounting the number of pixels in the punched-out damage location (assumed to be a circle of diameter 155 pixels, based on measurement), and after discounting the number of pixels outside of the region of interest or (ii) by the total number of ‘embolized’ pixels in each square. Normalization (i) is an index of absolute embolism amount while normalization (ii) is a relative index. Therefore, Option (i) (normalize by total number of lamina pixels in the square) is useful for providing a metric of the absolute amount of embolism events that occur in a given location. However, it is less suitable for comparing embolism rates among vein size classes, because it is biased towards having larger values in portions of the leaf that have more large veins (e.g. a midvein). Option (ii) (normalized by total number of pixels in the square) is useful for providing a metric of embolism rate, because it is not biased towards areas of the leaf that have more veins. However, it is less suitable for comparing embolism amounts and also may yield a noisy signal in squares where only a small number of pixels ultimately embolize. We had to use different T*x* values (50% vs. 1% in each case, i.e. T50 vs. T01) based on the typical trajectories of each metric. The ideal metric would actually be to normalize by the number of vein pixels in each square. We tried to do this by automatically segmenting each leaf sample (i.e. by identifying the vein and non-vein pixels) using Convolutional Neural Network (CNN) algorithms (LeafVeinCNN app in Matlab from Xu *et al.* 2021), but the CNN segmentations of vein pixels were not appropriate for this problem. This happened because the width of the segmented veins was somewhat dependent on image contrast, so that the total number of vein pixels (i.e. the denominator in the putative index) was more sensitive to algorithm parameters than to actual biology. Additionally, because of small shifts in the leaf position in the scanner (along with leaf shrinkage), it was not possible to easily mask the embolisms from the segmented vein mask. This was in principle doable but would have required a separate vein segmentation at each scan for each sample, which would have required a prohibitive amount of student labour and computation. We therefore settled on Option (i) and Option (ii) as reasonable compromises.

We did not calculate T01 with Option (i) because it was not considered biologically meaningful (indicating the early entry point to embolism); similarly, we did not calculate T50 with Option (ii) because it was never reached.

For each time series and each normalization method, we extracted T*x* metrics (time to embolism for a certain fraction *x* of pixels). For normalization (i) we used T01 (1% threshold), and for (ii) we used T50 (50% threshold). While T50 is a commonly used metric to compare overall hydraulic vulnerability across species and treatments, the T01 provides a better idea of how soon leaves start to embolize after the application of the treatment and was necessary as T50 was not reached for normalization (i) in many leaves (because many pixels were not vein pixels).

### Statistical analysis

To test whether the spatio-temporal patterns of embolism spread varied across venation (H1) and damage (H2) types, we compared T50 and the total fraction of leaf area embolized, and the colour-coded maps of embolism spread across each treatment. We also formally tested each hypothesis using a linear model of the form:

T50 or total fraction of embolized pixels ∼ species (pinnate/palmate) + damage type (midrib/lamina).

To investigate if embolism tends to spread faster near the herbivory-damaged areas (H3), linear models were fit as follows:

T50 or T01 ∼ species (pinnate/palmate) + damage type (midrib/lamina) + simulation treatment (squares from large/small veins or focal damage location) + species × experimental treatment + species × simulation treatment.

In all models, no random effect structure was used due to the low sample size and the balanced experimental design.

For H3, we also calculated *Z*-scores of each normalized embolism value in each leaf and at each time point, as the value in the focal damage location relative to the null distribution of normalized embolism values in either the lamina or midrib. This *Z*-score was not calculated for the control leaves, by definition. Values of the *Z*-score <0 indicate that the focal location (i.e. midrib or lamina-damaged area) is embolizing more slowly than the reference (i.e. non-damaged area around the midrib or around minor veins); values >0 indicate that the focal location is embolizing more rapidly than the reference. Values were taken to be statistically significant when the absolute *Z*-score exceeded 1.96, corresponding to a 95% confidence interval. No further statistical analysis was carried out due to the nature of the results obtained.

Image processing was carried out in MATLAB (version R2025a). Statistical analysis was carried out in R (version 4.4.3).

## Results

### Q1: Embolism did not spread faster in the pinnate network

**Figure 3:**
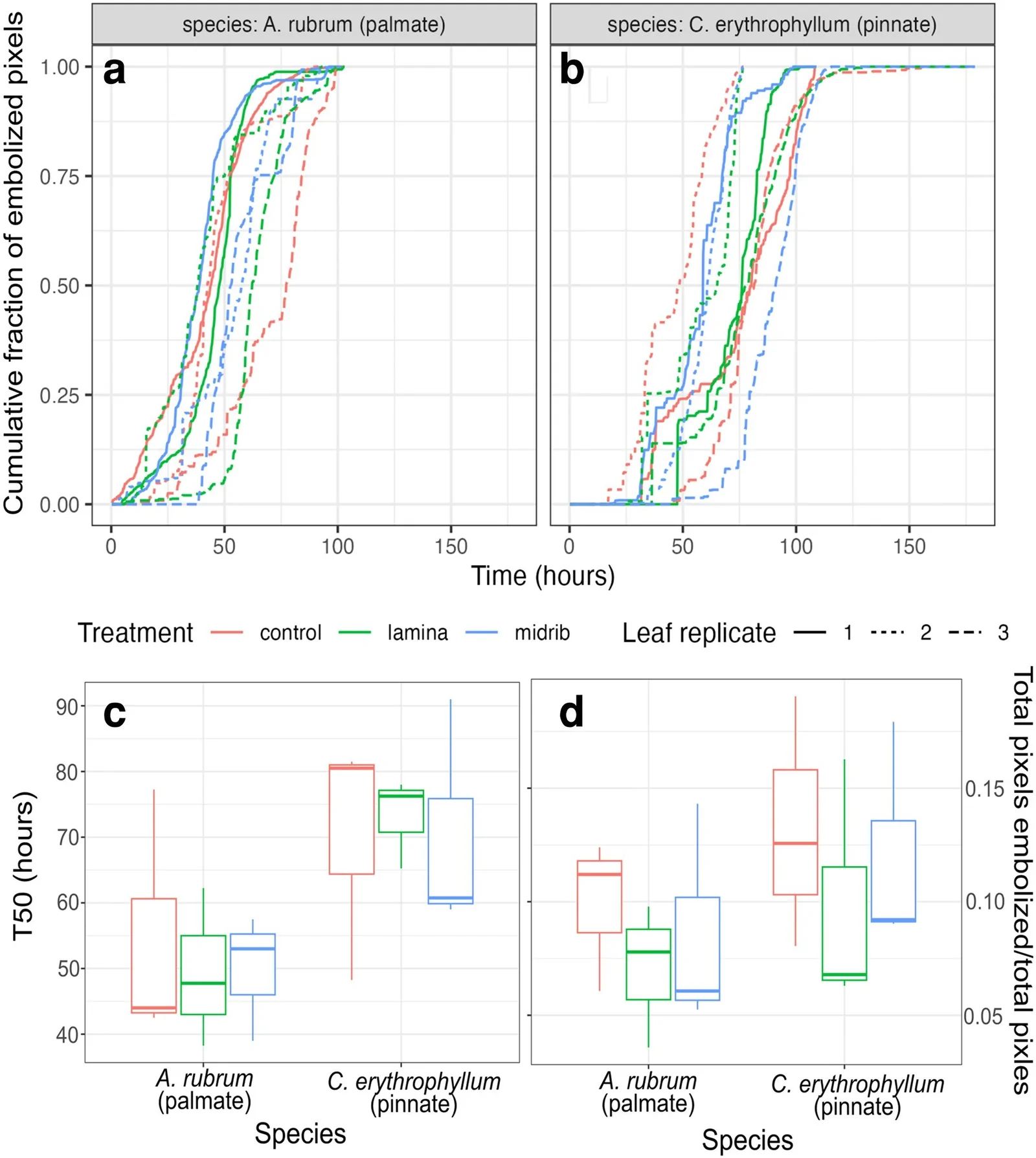
Observed whole leaf rates of embolization in response to drought and herbivory. (a) Whole leaf vulnerability curves for *A. rubrum* and *C. erythrophyllum* across the different damage treatments (control, lamina damage, and midrib damage). (b) Time to reach 50% of embolized pixels (T50) (normalized by total embolized pixels). (c) Total number of embolized pixels (normalized by leaf area).

The primary vein architecture (pinnate vs. palmate) influenced the spatio-temporal patterns of embolism spread under drought and herbivory damage (Fig. 3a and b; Supplementary Table S1). But, contrary to our expectation (H1), the palmate, rather than the pinnate leaf, reached T50 earlier in time (Fig. 3c) across all treatments (Supplementary Table S1, *P* < .01). In fact, in the control leaves of *A. rubrum* (palmate), the first embolism was recorded as soon as 360 minutes (6 hours) after the initiation of the dry-down experiment, whereas in *C. erythrophyllum* (pinnate), the first embolism was only observed after ∼11 hours. In the control treatment, the palmate leaves also reached hydraulic failure (i.e. maximum number of embolized pixels) around 12 hours faster than the pinnate leaves. However, no significant differences were found between species for the total number of embolized pixels (Fig. 3d; Supplementary Table S2, *P* = .14). These results suggest that having a larger number of low-resistance midrib veins in the palmate network can decrease, rather than increase, their tolerance to embolism formation and propagation.

### Q2: Damage to the midrib did not result in faster embolism spreading

Contrarily to H2, midrib damage did not result in consistently higher hydraulic vulnerability (i.e. lower T50) relative to the control or lamina treatments (Fig. 3). Although T50 was slightly lower in the midrib treatment for the pinnate (single midrib) species, these differences were not significant (Supplementary Table S1, *P* > .7). There was also high variation in the vulnerability curve parameters across replicates. For instance, some midrib- and lamina-damaged samples reached T50 before, while others reached it after the control samples (Fig. 3a and b). In both species, the total area of embolized pixels at the end of the experiment (Fig. 3d) did not significantly differ between the control and the damage (midrib or lamina) treatments (Supplementary Table S2, *P* > .2). Together, these results suggest that damage to the veins in drought-stressed leaves does not impact whole-leaf scale embolization rates, even when the damage is targeted at the midrib. This compartmentalization may enable continued leaf functioning after damage.

**Figure 4:**
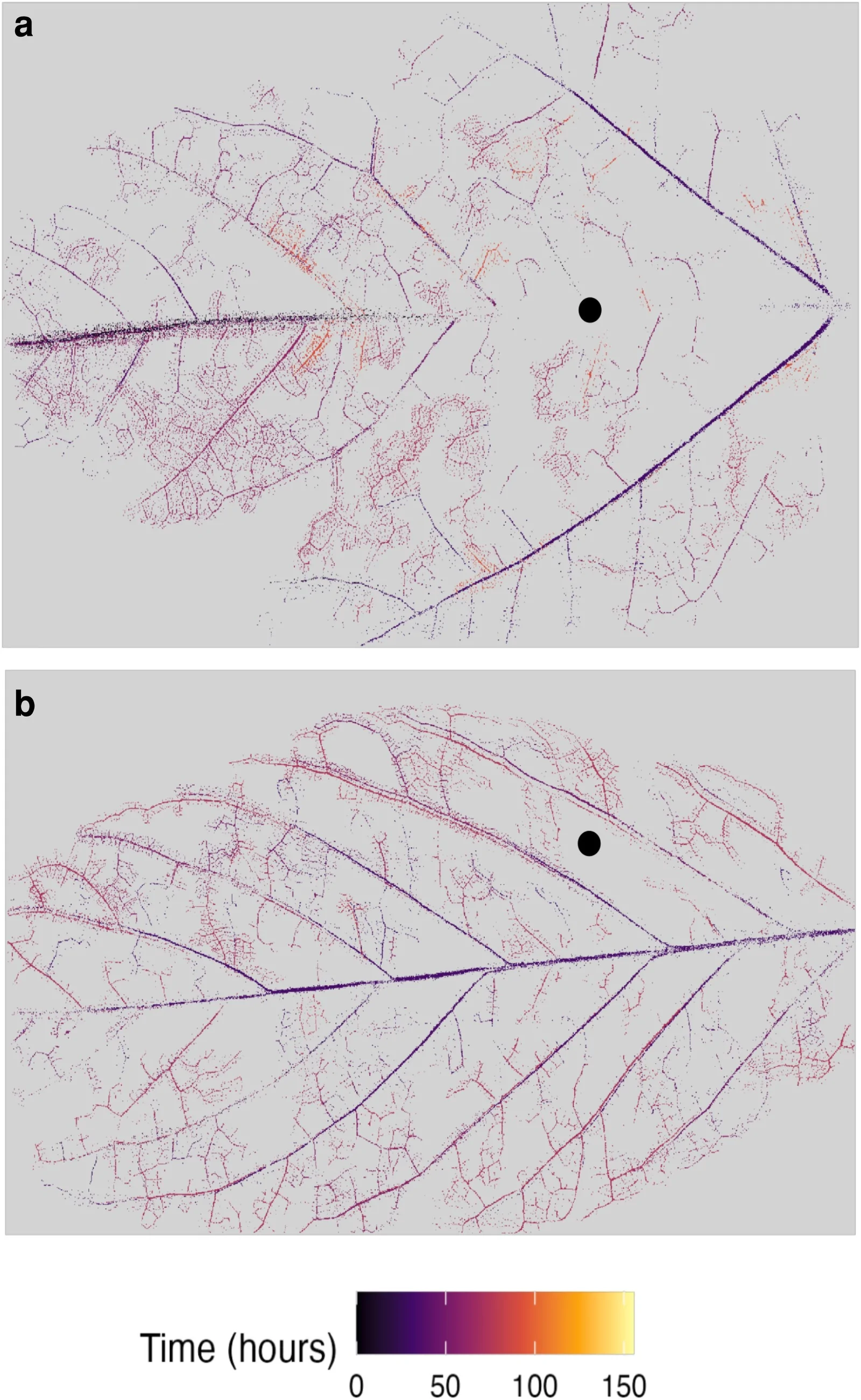
Example spatio-temporal maps of embolism propagation. Colour indicates the time of first embolization. The focal damage location is shown as a black dot. (a) *A. rubrum* (palmate), midrib treatment, second leaf. (b) *C. erythrophyllum* (pinnate) lamina treatment, second leaf. Line colour indicates the calculation basis; relative to ‘random_big’ indicates the distribution of embolisms in randomly selected midvein pixels, while ‘random_small’ indicates the distribution of embolisms in randomly selected minor vein pixels (compared with Fig. 2). Images of all other leaves are available as Supplementary Fig. S1.

Similarly, the spatial patterns of first embolism propagation were not strongly affected by the herbivory damage type (Fig. 4; Supplementary Fig. S1). Regardless of treatment, the sequence of initial embolization followed the usual pattern, with midrib embolizing first, then secondary veins, then the smaller vein sizes. At the whole leaf level, there was no evidence of earlier or additional embolism forming surrounding the damaged areas. In some cases, the area around the damaged midrib and lamina did not show embolism events (Fig. 4).

### Q3: Herbivory damage did not facilitate embolism spreading under drought

**Figure 5:**
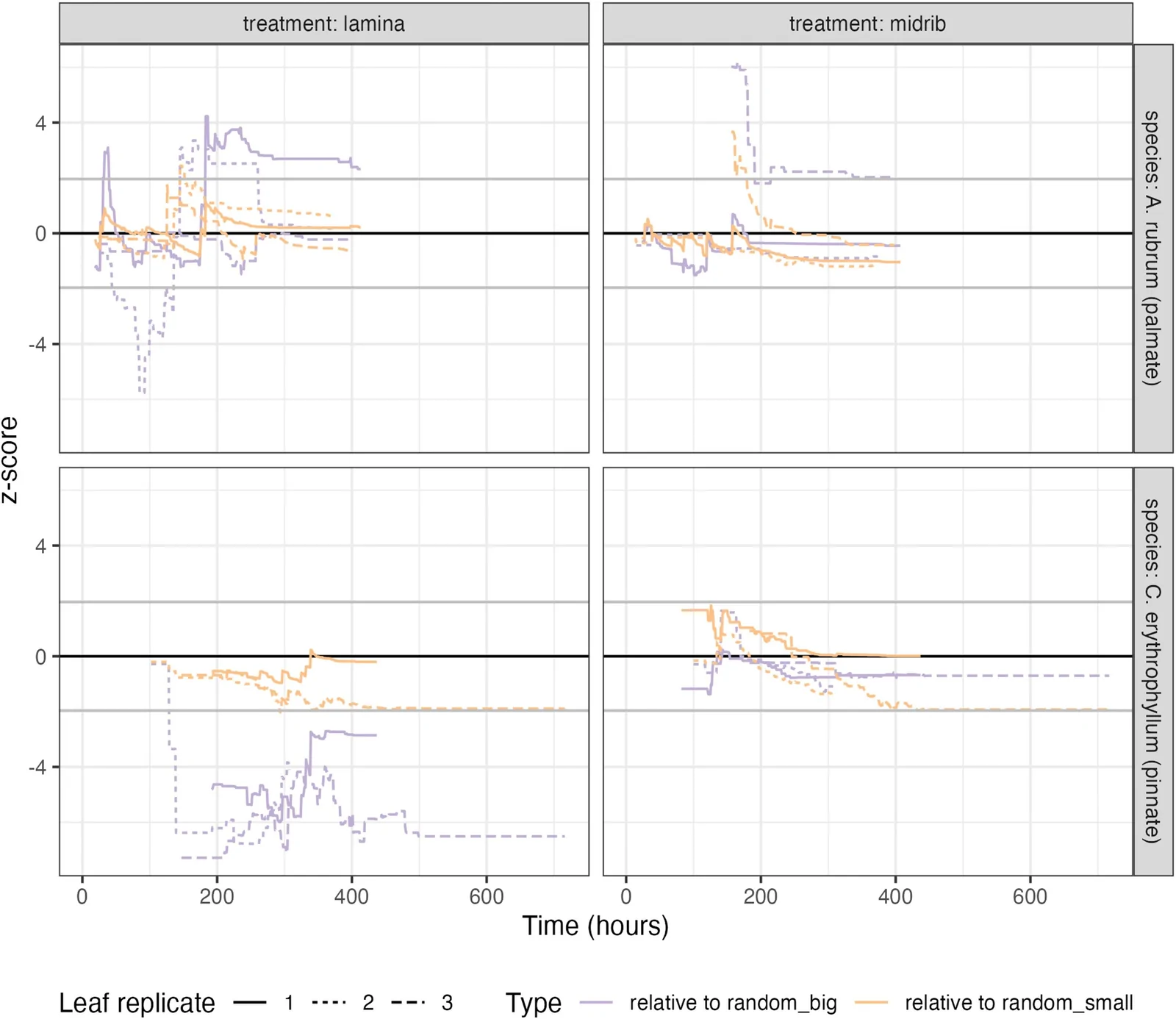
Interpretation of embolism propagation in terms of *Z*-scores, i.e. comparison of embolization in the focal damage region relative to the distribution of embolism in randomly selected midrib and minor vein locations. Values were normalized by the number of pixels in each square. Results are shown for 200 pixel square side lengths. The 95% confidence interval for a non-significant *Z*-value is shown in grey.

To further investigate whether the embolization rate was different near herbivory-damaged sites versus away from damaged areas (H3), we plotted the embolism temporal progression for the focal damaged areas relative to progressions for the non-damaged and randomly selected areas around the midrib and minor veins (Supplementary Fig. S2) and then compared their *Z*-scores (Fig. 5). Most *Z*-scores (across species and times) fell within the non-significant range. However, a few notable results were found. For the midrib treatment, embolism spreads faster near the focal damaged area only for one leaf replicate in the palmate species (Fig. 5). For the lamina treatment, embolism consistently spreads more slowly (rather than faster) near the damaged point than away from it, but only in the pinnate species (Fig. 5). Results were similar, regardless of the type of normalization used (Supplementary Figs S3 and S4).

To augment this *Z*-score analysis, a linear model of T01 (normalized by square area) indicated a positive effect of midrib/lamina damage treatment (*P* < .01) but no effect of species (*P* = .11) or simulation treatment (*P* > 0.4) (Supplementary Table S3 and Fig. S5); we therefore did not interpret interaction terms further. This result means that absolute rates of embolism appearance were ‘slower’ in damaged areas relative to controls. A linear model of T50 (normalized by total embolized pixels) indicated a negative effect of lamina treatment (*P* = .008) and no effect of simulation treatment (*P* > .05) or species (*P* > .05) (Supplementary Table S4 and Fig. S6). We therefore did not interpret interaction terms further. This result means that relative rates of embolism appearance were ‘faster’ in the lamina treatment but not impacted by species and not in locations closer to the actual lamina damage. Nevertheless, both the T01 and T50 linear models had low *R*^2^ (0.23–0.26), indicating high unexplained heterogeneity in responses among leaves. Overall, these results suggest that the punched holes did not facilitate new air-seeding events, as we expected (H3). Results were similar when varying the size of the squares (200 or 400 pixels) applied in the analysis (see GitHub repository).

## Discussion

A first striking finding from this study is that embolism does not seem to consistently spread outward from points of mechanical damage on the leaf. That is, the mechanical damage to the leaf does not lead to air-seeding of embolism. This was unexpected but ecologically interesting. One possible explanation is that such damage is rapidly sealed off, e.g. through production of leaf exudates. For example, xylem can seal off damaged conduits with tyloses (i.e. outgrowths of parenchyma cells into the xylem conduit lumen) and/or gels (also called gums, i.e. amorphous substances secreted by the parenchyma cells into the conduit lumen) to prevent further air spread throughout the venation network (Sun *et al.* 2008). While previous studies reported that the full occlusion of damaged conduits may take 1–2 weeks (Sack *et al.* 2008, Sun *et al.* 2008), the initial response is immediate, and the partial sealing (e.g. incompletely expanded tyloses) might be enough to prevent air-seeding events around damaged areas. Although the two species investigated here do not possess latex, this compound could also be involved in a faster response to damaged veins (A. Agrawal, 2025, personal communication). Presumably, faster occlusion responses to herbivory would be highly adaptive given the prevalence of mechanical damage to leaves under natural conditions. Interestingly, Guan *et al.* (2021) did observe embolism propagating from damaged minor veins (note that larger veins were not investigated in their study), so there may be substantial heterogeneity in responses among species. Future studies should investigate a larger number of species and include anatomical analyses to assess whether, and how fast after damage, veins can be sealed and how such sealing affects drought vulnerability.

A second alternative explanation for the absence of additional embolism around the damaged leaf areas is that cut-open xylem conduits may remain mostly filled with water even after the damage. When xylem conduits are cut open in air, there is an expectation that they would become immediately filled with gas, as the water inside these damaged conduits could be quickly withdrawn into neighbouring intact conduits due to its negative pressure (Wheeler *et al.* 2013). Because embolism propagation is largely known to occur from already embolized to non-embolized neighbouring conduits, the areas surrounding the damaged and gas-filled conduits could undergo higher embolism rates as the leaf dries out (Guan *et al.* 2021). However, recent studies (Miranda *et al.* 2026) with stem xylem have indicated that the first assumption may not always be correct and xylem sap may not be drained immediately out of damaged conduits, particularly in fairly well-hydrated samples. Since our samples were damaged at the beginning of the dry-down experiment, when leaves were fully hydrated, the xylem tension inside the conduits may not have been sufficiently large to aspirate air into the open conduits (Wheeler *et al.* 2013, Miranda *et al.* 2026), so damaged conduits may not have become a significant source of embolism propagation. This suggests that although punch-type herbivory damage may not have a significant hydraulic impact on initially well-hydrated leaves, it may lead to faster embolism spreading if the herbivory damage affects leaves that are already drought-stressed (i.e. under higher xylem tension). Future studies could easily test this idea by damaging leaves at different water potentials and examining the herbivory damage impacts on the spatio-temporal patterns of embolism propagation.

A third alternative explanation is that punching the holes could have caused immediate embolism in those areas, which may not have been captured by the scanner. We cannot rule this possibility out in the data. However, it is also possible, and perhaps likely, that the punch did not cause an initial embolism. This is because in the midvein punch treatments we still saw embolism occur in the midvein in our imaging, and also this embolism occurred first in the midvein rather than in other locations.

Another striking finding from this study is the substantial heterogeneity in the spatial and temporal spread of embolism among leaves, independent of species or damage treatment. This may suggest stochasticity in the embolism spread process or real variation in pre-existing damage within leaves leading to deterministic variation in the spread process, e.g. (Avila *et al.* 2022). While we selected leaves to be apparently damage-free, we cannot distinguish between these possibilities. The main interpretation is that apparently similar leaves may experience highly different outcomes after damage. Consequently, future studies must use larger sample sizes to better detect species responses to different herbivory damage treatments.

Our results also call into question whether the number of embolized pixels is indeed a reliable proxy for per cent loss of conductivity, as it has been routinely used in optical vulnerability analysis (Brodribb *et al.* 2017). Presumably some pixels are more important than others—if the midrib is disconnected early on, one might imagine that water flow would necessarily be interrupted. The only alternatives are that conductance remains high in other locations of the leaf due to high leaf capacitance or due to the continued existence of outside-xylem flow pathways within the leaf. Ruling out the leaf capacitance possibility is difficult in dry-down experiments like ours where leaves are not able to be studied for extended periods of time at intermediate water potentials. This study does suggest that xylem damage, both directly from punching and indirectly from potential air-seeding, surprisingly does not substantially affect the embolization process. As such, these results align closely with Scoffoni *et al.* (2017a), which has focused on the importance of outside-xylem processes for conductance. Nevertheless, more detailed investigation of underlying processes during embolism spread would be needed to provide further clarity.

The study has several limitations that make the findings discussed above more exploratory rather than definitive. First, we cannot confidently make inferences about palmate versus pinnate architecture, because we had resources available to study only one species of each architecture. Second, we were not able to obtain enough water potential data to compare P50 across species and treatments. Nevertheless, the T01/T50 metrics studied here provide complementary insights and critically were also measurable across different locations in the leaf. Third, the mechanical simulation of herbivory employed here may be an incomplete proxy for insect herbivory. Differences may arise due to the absence of chemical and mechanical cues sensed by plants in response to real herbivores, which in turn may impact the induction of plant signalling and defence pathways (Wu and Baldwin 2010, Waterman *et al.* 2019). We were not able to assess these effects due to the scope of this study. Fourth, the segmentation of embolism in scanned images varied in its precision among leaves (some have more noisy pixels around embolized veins than others), mostly due to shrinkage issues as the leaves dried out. While this variation could affect the total pixels analysis (Supplementary Table S2), all other analyses are normalized per leaf and should remove this bias. Nonetheless, motion compensation algorithms should be explored in future studies using the optical method to potentially correct shrinkage issues, particularly when constructing vulnerability curves for whole leaves (rather than small leaf sections). Lastly, available water storage in the stem and total rate of transpiration from the non-focal leaf could have influenced the overall rate of embolism progression in the focal leaf. We do not have any data on the relevant traits (e.g. rate of water loss for the leaves inside vs. outside scanner, branch capacitance, etc.) to assess these possibilities. Nonetheless, we have some confidence that any such biases are at least consistent within and across species, thus allowing proper comparisons of vulnerability both across treatments and species. All branch segments used in this study had similar lengths (∼1 m), basal diameter (∼1–1.5 cm), and number of leaves between the same species replicates. However, *C. erythrophyllum* had more leaves on the branch than *A. rubrum*, but we do not have quantitative data confirming the difference between the two, only a picture for representation (Supplementary Fig. S7). The positioning of each branch in the scanner was also similar, i.e. with the same number of leaves placed under the scanner top. All experiments were conducted in the same room with controlled temperature, and sampled branches were rehydrated at night in the dark with black trash bags placed over them.

Overall, our study advances understanding of the combined impact of drought and herbivory damage on leaf hydraulic functioning. It shows that, at least in some species, herbivory-damaged leaves are not more vulnerable to embolism formation and propagation under drought, even when the herbivory damage impacts the largest veins in the network. While the exact mechanisms are unclear and deserve further investigation, it suggests that leaf responses to herbivory and to drought may be separate, rather than coupled, axes of variation. Plants may have evolved different approaches to the challenges of dealing with herbivory (point damage) versus drought (systemic damage).

## Supplementary Material

plag033_Supplementary_Data

## Data Availability

Datasets, image analysis code, and statistical analysis code are available at https://github.com/bblonder/embolism_spatial_spread. They are archived on Zenodo at https://zenodo.org/records/21283113.
