# Supplementary material for: Embolism does not spread outwards from simulated herbivory damage sites in leaves
Source: AoB Plants. 2026 Aug 24;18(4):plag033. doi: 10.1093/aobpla/plag033 (PMC13501410; doi:10.1093/aobpla/plag033)
Supplement: Supplementary file 1 [file plag033_supplementary_data.pdf]

Supporting Information

Table S1.

Regression table for whole-leaf T50.

	time_hours		
Predictors	*Estimates*	*CI*	*p*
(Intercept)	52.39	38.19 – 66.59	**<0.001**
species [C. erythrophyllum (pinnate)]	19.89	5.69 – 34.09	**0.009**
treatment [lamina]	-1.04	-18.43 – 16.35	0.900
treatment [midrib]	-2.29	-19.68 – 15.10	0.782
Observations	18		
R^2 / R^2 adjusted	0.394 / 0.264		

Table S2.

Regression table for fraction of pixels embolized.

	**n_pixels_embolized/px_mask**		
Predictors	*Estimates*	*CI*	*p*
(Intercept)	0.10	0.06 – 0.14	**<0.001**
species [C. erythrophyllum (pinnate)]	0.03	-0.01 – 0.08	0.145
treatment [lamina]	-0.03	-0.09 – 0.02	0.236
treatment [midrib]	-0.01	-0.07 – 0.04	0.627
Observations	18		
R^2 / R^2 adjusted	0.219 / 0.052		

Table S3.

Regression table for T01 (normalized by total # of available pixels in each square). Results are for a square side of 200 pixels.

	time_hours		
Predictors	*Estimates*	*CI*	*p*
(Intercept)	31.51	15.17 – 47.85	**<0.001**
species [Com_ery]	17.63	-4.50 – 39.76	0.118
treatment [L]	3.92	1.80 – 6.04	**<0.001**
treatment [M]	3.73	1.48 – 5.97	**0.001**
simulation treatment [random_big]	-6.92	-23.22 – 9.38	0.405
simulation treatment [random_small]	3.79	-12.50 – 20.08	0.648
species [Com_ery] x treatment [L]	-0.75	-3.74 – 2.23	0.621
species [Com_ery] x treatment [M]	8.58	5.49 – 11.66	**<0.001**
species [Com_ery] x simulation treatment [random_big]	-6.86	-28.93 – 15.20	0.542
species [Com_ery] x simulation treatment [random_small]	-4.40	-26.46 – 17.66	0.696
Observations	3430		
R^2 / R^2 adjusted	0.230 / 0.228		

Table S4.

Regression table for T50 (normalized by total # of embolized pixels in each square). Results are for a square side of 200 pixels.

	time_hours		
Predictors	*Estimates*	*CI*	*p*
(Intercept)	46.08	31.88 – 60.27	**<0.001**
species [C. erythrophyllum (pinnate)]	19.71	-0.36 – 39.78	0.054
treatment [lamina]	-2.68	-4.68 – -0.69	**0.008**
treatment [midrib]	-1.80	-3.81 – 0.20	0.077
simulation treatment [random_big]	-0.22	-14.37 – 13.92	0.975
simulation treatment [random_small]	6.55	-7.59 – 20.70	0.364
species [C. erythrophyllum (pinnate)] x treatment [lamina]	-2.75	-5.57 – 0.07	0.056
species [C. erythrophyllum (pinnate)] x treatment [midrib]	11.25	8.42 – 14.08	**<0.001**
species [C. erythrophyllum (pinnate)] x simulation treatment [random_big]	-14.04	-34.05 – 5.96	0.169
species [C. erythrophyllum (pinnate)] x simulation treatment [random_small]	-3.30	-23.30 – 16.71	0.747
Observations	3591		
R^2 / R^2 adjusted	0.265 / 0.263		

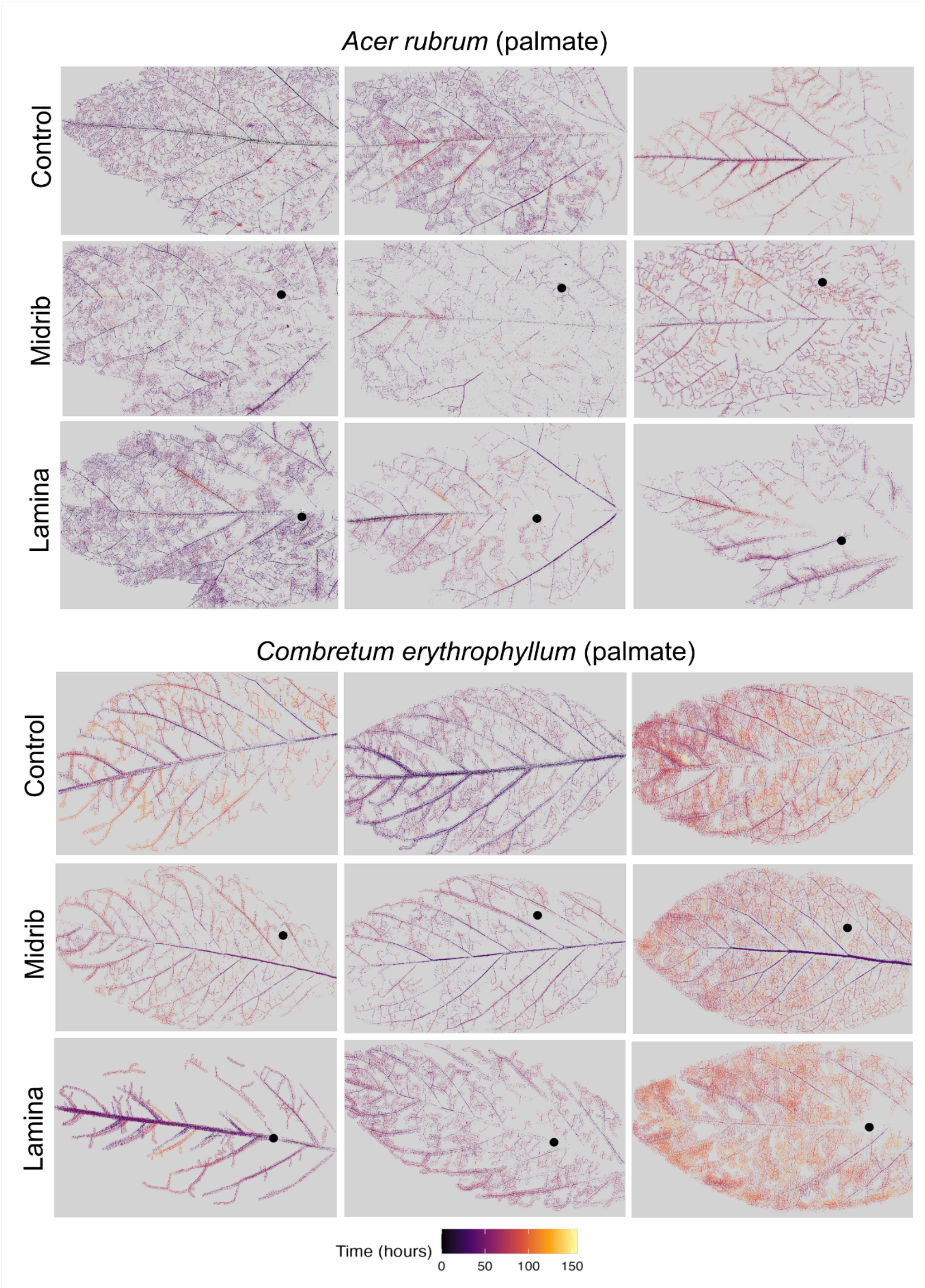

Figure S1.

Spatio-temporal maps of embolism propagation in *A. rubrum* (palmate) and C. *erythrophyllum* (pinnate) leaves in the control and damage (midrib and lamina) treatments (rows), with three leaf replicates per treatment (columns). Damage locations are shown as black circles.

ALT TEXT: Embolism maps for all 18 sampled leaves showing where an embolism occurred within the veins of the sampled leaf over time.

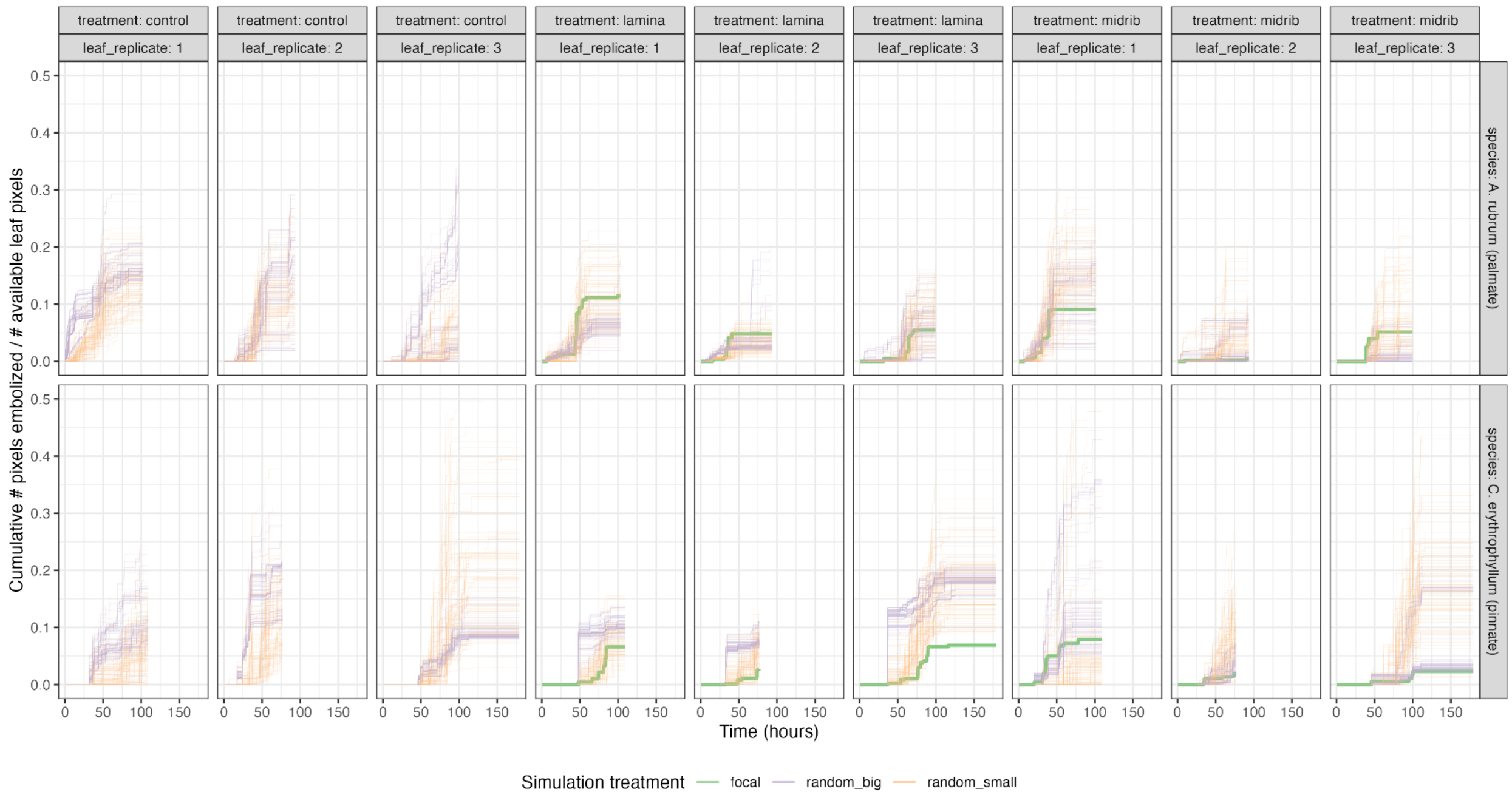

Figure S2.

Embolism progression in square regions (200 pixel side length) for each species and damage treatment. Progression in the focal region is shown in green; in the midrib (‘random_big’) in purple, and in the minor veins (‘random_small’) in orange.

ALT TEXT: Graphs for all 18 sampled leaves displaying their embolism progression over time.

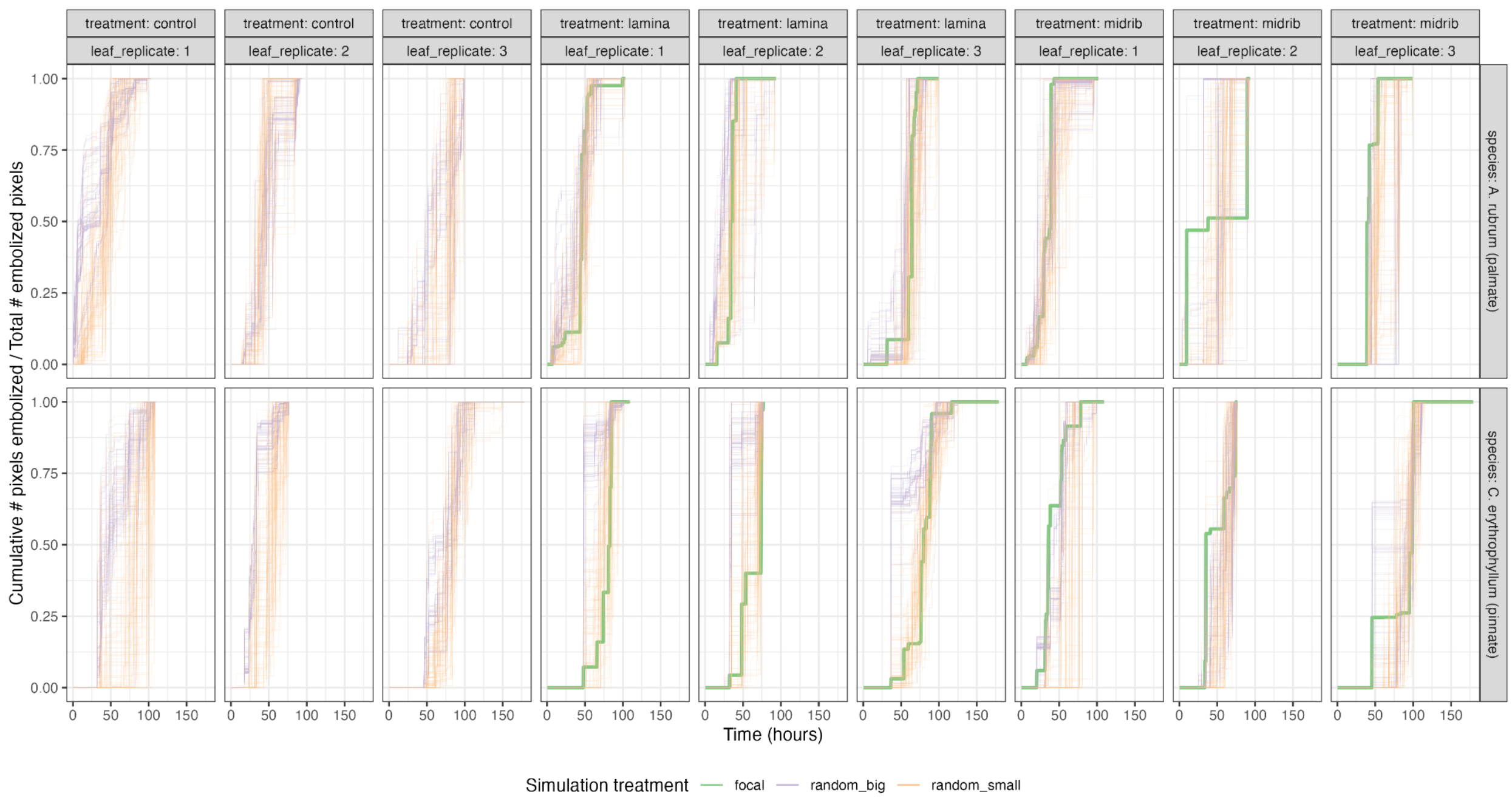

Figure S3.

Data in **Figure S2**, but normalized by maximum number of pixels embolized in each square.
ALT TEXT: Graphs for all 18 sampled leaves displaying their normalized embolism progression over time

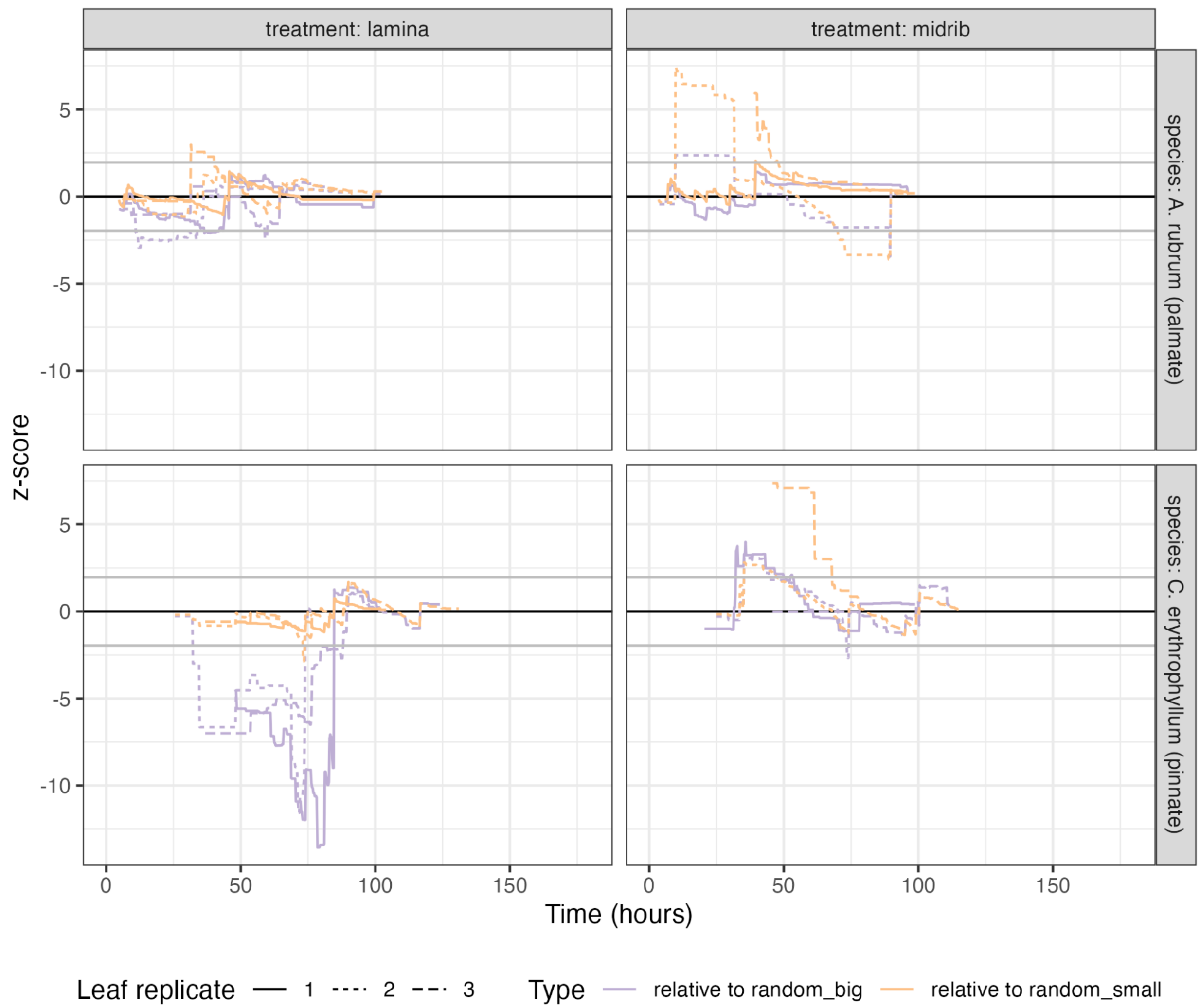

Figure S4

Data in **Figure 5**, but normalized further by the total # of pixels embolized within each square.
ALT TEXT: Graphs comparing normalized z-scores for both species, treatments, and replicates over time.

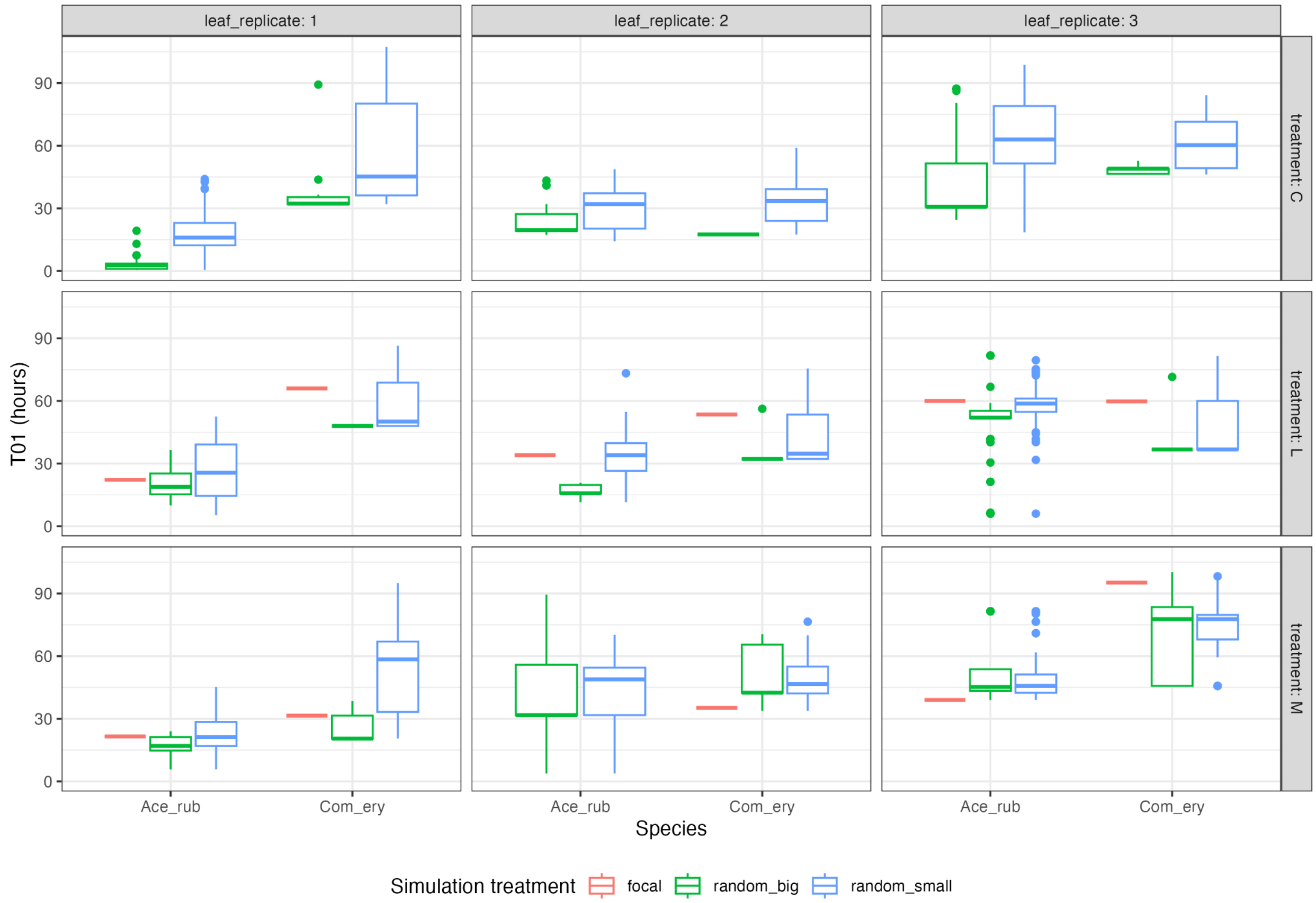

Figure S5.

T01 (normalized by available pixels within each square) values for each species, treatment, and leaf combination. Results are shown for a 200 pixel square side length.

ALT TEXT: Box plots showing all sampled leaves with T01 and the simulation treatments.

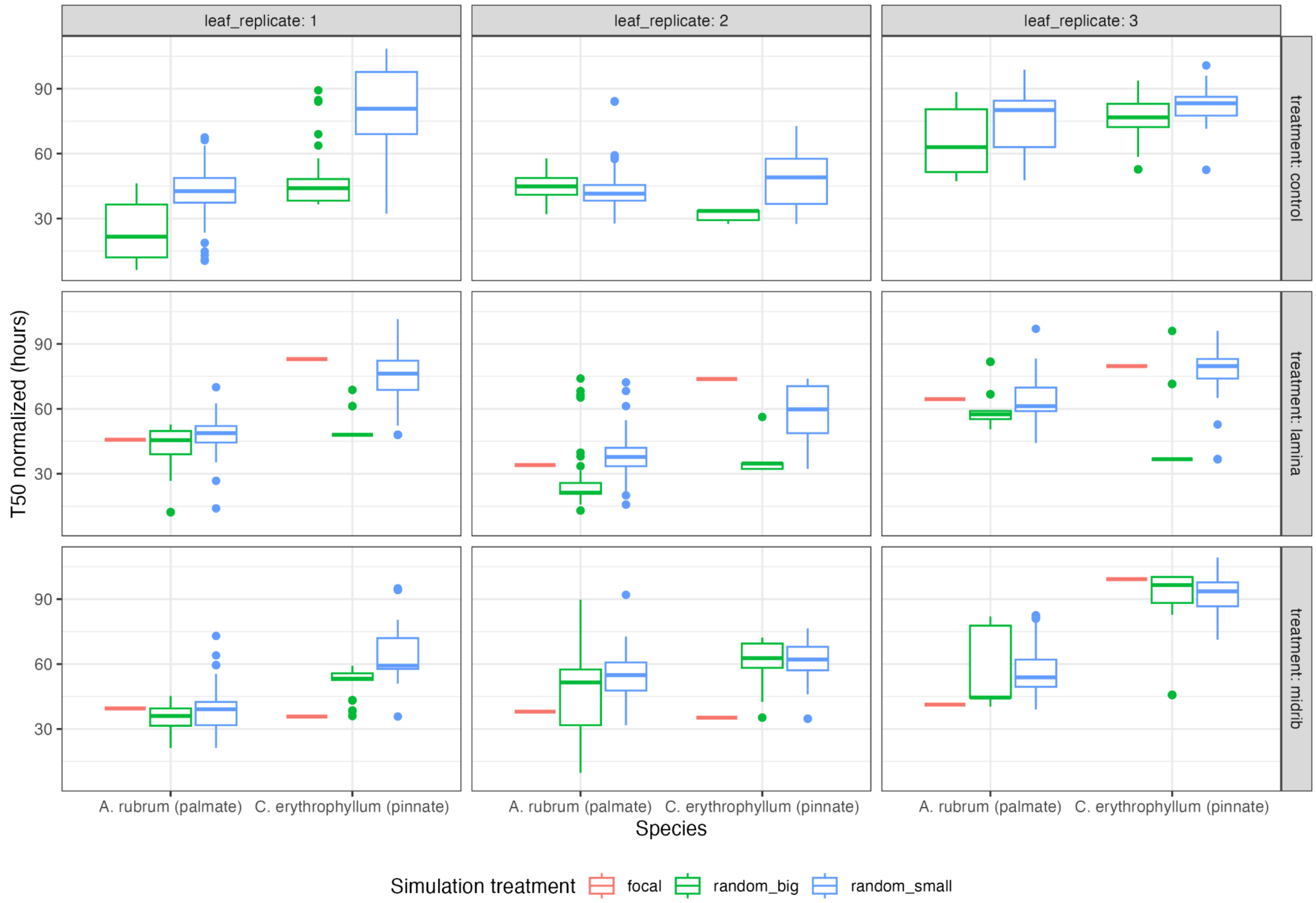

Figure S6.

T50 (normalized by total # of embolized pixels within each square) values for each species, treatment, and leaf combination. Results are shown for a 200 pixel square side length.
ALT TEXT: Box plots showing all sampled leaves with T50 and the simulation treatments.

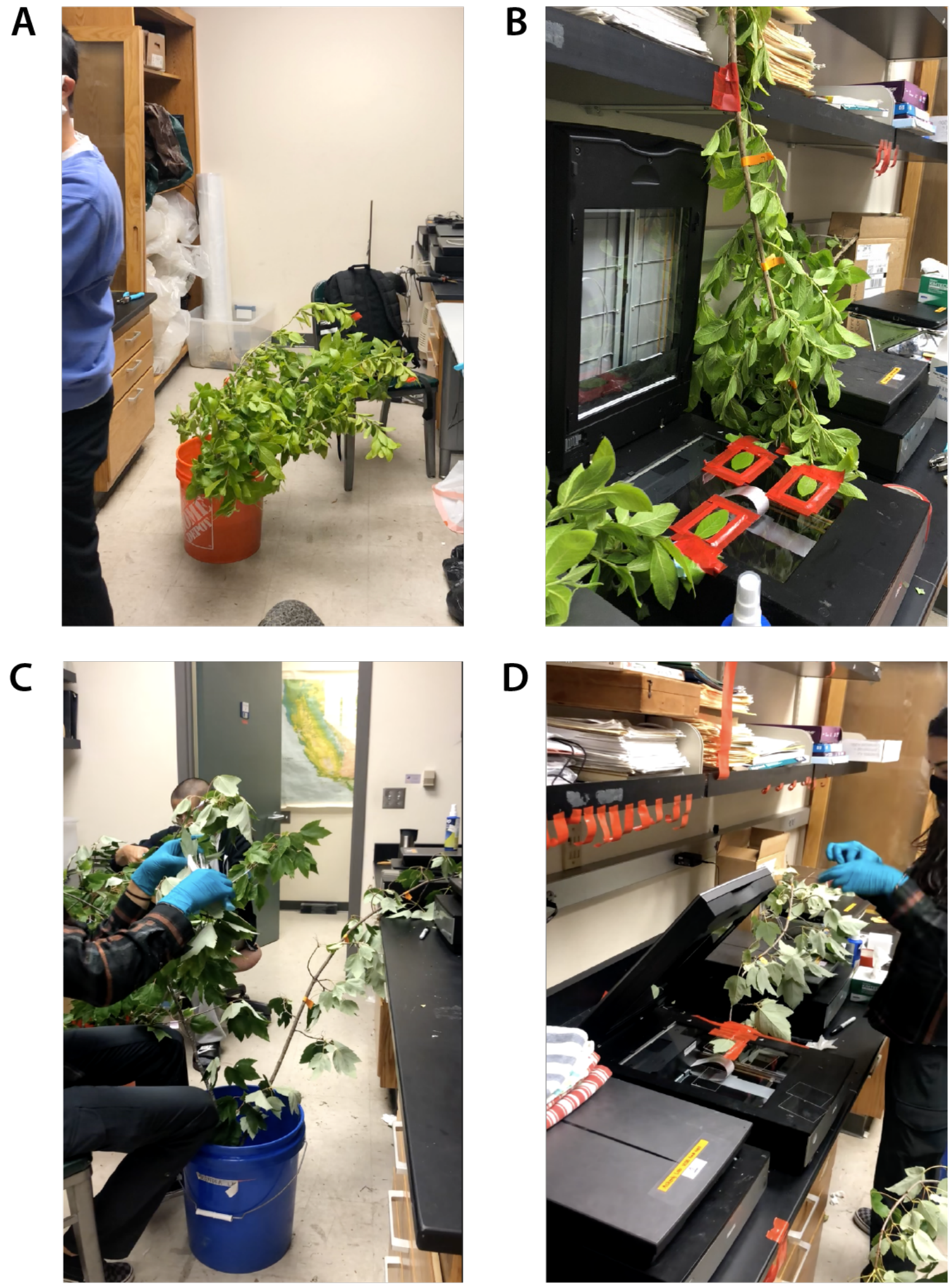

Figure S7.

Photographs of sampled branches being rehydrated and placed on a scanner. A-B show *Combretum erythrophyllum*, and C-D show *Acer rubrum*.

ALT TEXT: Four photos of branches of two different species, in buckets and on a scanner.
